# Synthesis and Characterization of the Inclusion Complex of β-cyclodextrin and Azomethine

**DOI:** 10.3390/ijms14023671

**Published:** 2013-02-07

**Authors:** Kavirajaa Pandian Sambasevam, Sharifah Mohamad, Norazilawati Muhamad Sarih, Nor Atiqah Ismail

**Affiliations:** Department of Chemistry, Faculty of Science, University of Malaya, Kuala Lumpur 50603, Malaysia; E-Mails: kavirajaa@live.com (K.P.S.); nmsarih@um.edu.my (N.M.S.); atiqahtariqa@yahoo.com (N.A.I.)

**Keywords:** β-cyclodextrin, azomethine, Schiff bases, inclusion complex

## Abstract

A β-cyclodextrin (β-Cyd) inclusion complex containing azomethine as a guest was prepared by kneading method with aliquot addition of ethanol. The product was characterized by Fourier Transform Infrared (FTIR) spectrometer, ^1^H Nuclear Magnetic Resonance (^1^H NMR) and Thermogravimetric Analyzer (TGA), which proves the formation of the inclusion complex where the benzyl part of azomethine has been encapsulated by the hydrophobic cavity of β-Cyd. The interaction of β-Cyd and azomethine was also analyzed by means of spectrometry by UV-Vis spectrophotometer to determine the formation constant. The formation constant was calculated by using a modified Benesi-Hildebrand equation at 25 °C. The apparent formation constant obtained was 1.29 × 10^4^ L/mol. Besides that, the stoichiometry ratio was also determined to be 1:1 for the inclusion complex of β-Cyd with azomethine.

## 1. Introduction

The studies on supramolecular chemistry give a broad idea of intermolecular interactions where covalent bonds are not likely to form between the interacting species. Thus, most of this interaction has been performed by host-guest interaction. Among the host molecules, cyclodextrin seems to be the most promising to form inclusion complexes, especially with various guest molecules with suitable polarity and dimensions [1,2].

Cyclodextrin molecules are cyclic oligosaccharides made up of six to twelve α-d-glucopyranose monomers, which are connected at 1 and 4 carbon atoms. Cyclodextrins with six to eight α-d-glucopyranose units are denoted as α-, β- and γ-Cyclodextrins respectively. Among these various types of cyclodextrins, α-cyclodextrin is not suitable for many drugs and γ-cyclodextrin is expensive. β-cyclodextrin is widely used because it is readily available, and its cavity size is suitable for a wide range of guest molecules. In general, the special characteristic of cyclodextrins is the ability to form an inclusion complex with various organic molecules through host-guest interaction with the interior cavity that provides hydrophobic environment to trap an apolar pollutant [3]. The inclusion complex of these host–guest systems occurs through various interactions, such as hydrogen bonding, van der Waals interaction, hydrophobic interactions and also electrostatic attraction [2], where the described types of bonding would alter the photochemical and photophysical properties of the guest molecules. Thus, the physical, chemical and biochemical properties of guest molecules will be modified and the application criteria of those guest molecules also can be improved [4].

**Figure 1:**
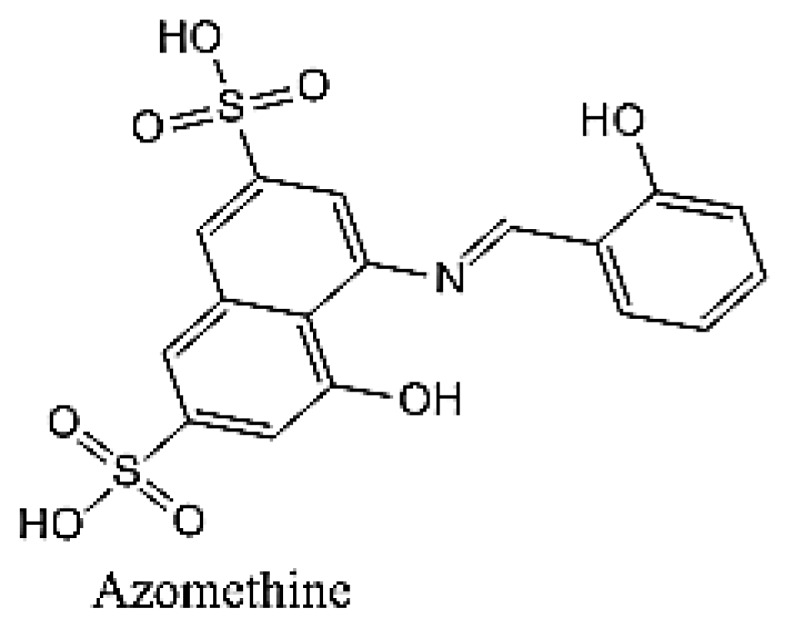
Chemical structure azomethine.

So far, various kinds of guest molecules such as drugs, steroids, ionic liquids and dyes were used as host-guest interaction to change the properties of the guest molecules into the desired form. Schiff bases are compound with a functional group that contains a carbon nitrogen double bond with the nitrogen atom connected to an aryl or alkyl group. Azomethine is one examples of a Schiff based compound (Figure 1) [5]. According to the previous research [6], azomethine has shown some applications in therapeutic applications to exert anti-tumor activity. In addition, Smith and Chen [7] have demonstrated that azo compounds can be used as chromophores and metalochromic agents. Azomethine (Schiff based) compounds are widely used due to the good mechanical strength [8], attractive thermal stability [9], nonlinear optical materials, optical interconnect, oscillator, amplifier, frequency converter [10] and liquid crystal thermosets (LCTs) [11]. However, poor solubility of these azomethines in organic solvents limits their applications in various fields. Nepal *et al.* [12] have introduced β-cyclodextrin to the poly (azomethines) to improve the solubility and processability of Schiff bases for various types of applications.

In this study, we have synthesized a novel inclusion complex of β-cyclodextrin and azomethine, which was not reported elsewhere. We believe this work would be the initial step to investigate the interaction feasibility of β-cyclodextrin and azomethine. Thus, we have chosen simple, highly sensitive and selective methods for the characterization of inclusion complexes such as Fourier transform-infrared (FTIR) spectrometry, proton nuclear magnetic resonance (^1^H NMR) spectrometry, thermogravimetric analysis (TGA), and ultraviolet-visible (UV-Vis) spectrometry [13].

## 2. Results and Discussion

### 2.1. Characterizations

**Figure 2:**
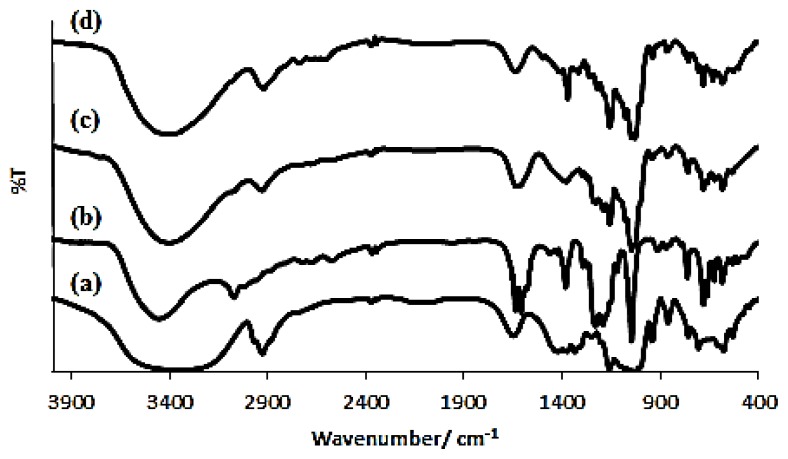
The Fourier transform-infrared (FTIR) spectra of (**a**) β-cyclodextrin; (**b**) azomethine; and (**c**) physical mixture of azomethine and β-Cyd; (**d**) inclusion complex of azomethine-β-Cyd.

FTIR is a very useful tool to prove the existence of both guest and host molecules in their inclusion complexes [14]. Figure 2 shows the FTIR spectra for the (a) β-cyclodextrin, (b) azomethine and (c) physical mixture of azomethine and β-Cyd and (d) inclusion complex of azomethine-β-cyclodextrin. The spectrum for the inclusion complex looks almost similar to the pure β-cyclodextrin. It indicates the formation of the inclusion complex, similar to a phenomenon observed by Li *et al.* [15]. Besides that, a broad hydroxyl band of pure β-cyclodextrin at 3370.72 cm^−1^ was found to be narrowed in the FTIR spectrum of the inclusion complex which is a good indication of the formation of the inclusion complex; this is a common phenomenon observed by many researchers in synthesizing the inclusion complex between β-cyclodextrin (host) and a guest molecules [15–18].

The frequencies for β-Cyd observed at 3370.72 cm^−1^, 2928.53 cm^−1^, 1157.84 cm^−1^, and 1029.24 cm^−1^ which corresponds to the symmetric and antisymmetric stretching of ν[OH], ν[CH_2_], ν[C–C] and bending vibration of ν[O–H] respectively. Meanwhile the frequencies for azomethine were recorded at 3449.28 cm^−1^, 1608.52 cm^−1^ and 681.39 cm^−1^ for the respective functional groups such as ν[OH], ν[C=C] and vibrational stretching of ν[=C–H]. Tables 1 and 2 have shown the difference in frequencies between β-Cyd and the inclusion complex; and between azomethine and inclusion complex respectively.

Both tables show some increase and decrease in intensity changes, Δδ. The increment is due to the insertion of the benzene part ring into the electron rich cavity of β-cyclodextrin and will increase the density of electron cloud, which will lead to the increase in frequency [19]. The decrease in the frequency between the inclusion complex and its constituent molecule is due to the changes in the microenvironment which lead to the formation of hydrogen bonding and the presence of van der Waals forces during their interaction to form the inclusion complex [20]. On the other hand, the FTIR spectrum of physical mixtures imitated the characteristic peaks of β-Cyd and azomethine, which can be regarded as a simple superimposition of those host and guest molecules. Thus, the FTIR spectra significantly proves the formation of the azomethine-β-Cyd inclusion complex.

Insertion of a guest molecule into the hydrophobic cavity of the β-cyclodextrin will result in the chemical shift of guest and host molecules in the NMR spectra. In general, large chemical shifts will be observed at H3 and H5, which are located in the inner cavity of cyclodextrin due to the inclusion phenomena [21]. The chemical shift change (Δδ) is defined as the difference in chemical shift change, positive sign means a downfield shift and negative sign means an upfield shift [22]. Table 3 exhibits the peak assignments for β-Cyd and the inclusion complex of azomethine-β-Cyd.

**Figure 3:**
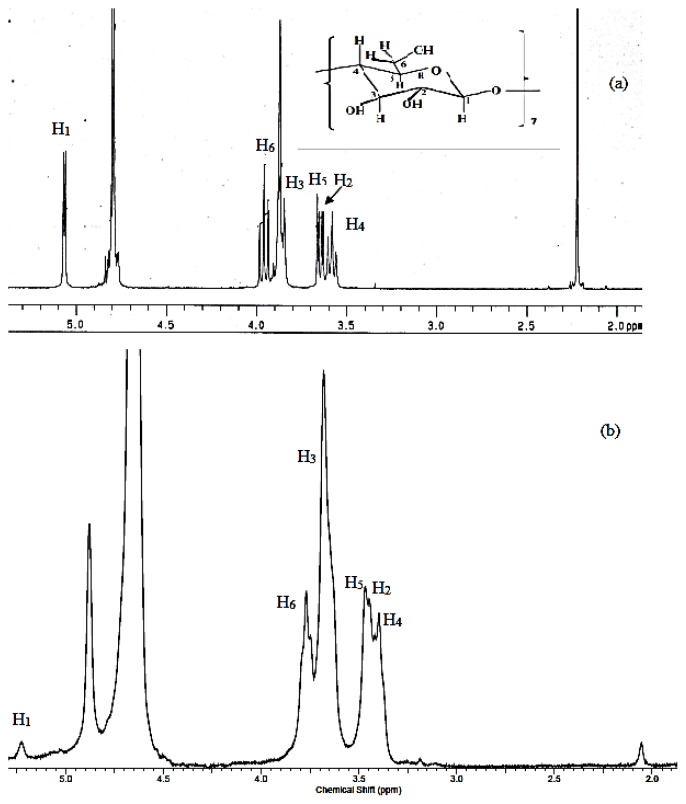
^1^H NMR spectra of (**a**) β-Cyd and (**b**) inclusion complex.

Figure 3 shows, the ^1^H NMR spectra of β-Cyd and inclusion complex protons. In this study only β-Cyd and inclusion complex protons could be discussed because too many peaks were overlapped in 7.0–8.2 ppm region of azomethine indicating the aromatic ring [3]. Table 3 shows some chemical shifts observed for H1, H2, H3, H4, H5 and H6. However, the chemical shifts for protons such as H3 and H5 (located inside the cavity of β-Cyd) are slightly higher compare to other protons, which are located at the exterior side of the cavity. Therefore, this shift provides the indication for the formation of the inclusion complex between β-Cyd and azomethine [23–28].

**Figure 4:**
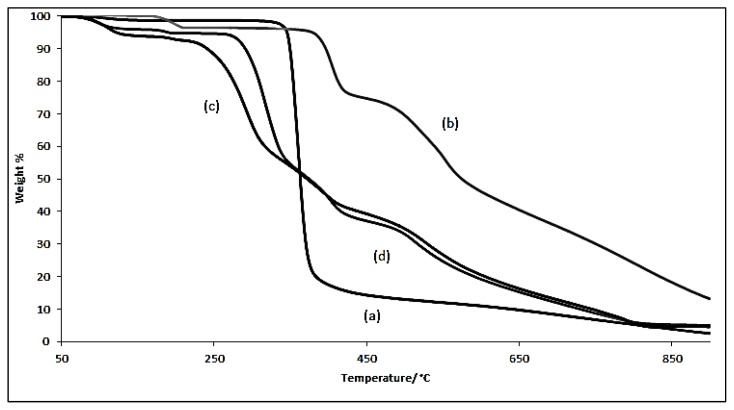
TGA for (**a**) β-cyclodextrin; (**b**) azomethine; (**c**) inclusion complex; (**d**) physical mixture.

Thermogravimetric analysis (TGA) will be done on samples to identify the changes in weight percent with respect to temperature change. TGA was performed on pure β-Cyd, azomethine and their inclusion complex. TGA results plotted in Figure 4 in the temperature range of 50 °C to 850 °C. β-Cyd exhibits two separate weight losses due to loss of water molecules at 99.3 °C which was located in the cavity of β-Cyd and followed by the decomposition of macrocycles at 343 °C [29]. Meanwhile azomethine exhibited weight losses at 170 °C and 380 °C which were due to the degradation of benzyl part and–CH=N– respectively. Then the inclusion complex underwent weight losses in three stages and lost 95% of its original weight at 809 °C. The first stage is due to the dehydration of water molecules and the second stage is due to the decomposition of β-Cyd and the third stage probably due to the decomposition of azomethine. In comparison, to confirm the formation of the inclusion complex, thermal analysis has been done on physical mixture of β-Cyd and azomethine. The first weight loss for the inclusion complex starts at 71 °C whereas it starts at 95 °C for the physical mixture which is almost similar to the water loss of β-Cyd. It means the formation of inclusion complex has changed the thermal degradation properties of β-Cyd and azomethine. In addition, the second decomposition for β-Cyd was around 343 °C but for the inclusion complex it has been decreased to 225 °C. This phenomenon suggests that formation of inclusion complex decrease the thermal stability of β-Cyd [24].

### 2.2. Spectroscopic Studies

#### 2.2.1. Effect of pH

The effect of pH has been studied to determine the optimum pH for inclusion formation between β-Cyd and azomethine. pH 7 was chosen to be the optimum pH for this inclusion complex due to the highest absorbance at 236 nm. This is a common phenomena as observed by Cesla *et al.* where derivative of sulphonated azo dyes like azomethine can form highly stable inclusion complex with β-Cyd at neutral pH [30].

#### 2.2.2. Absorption Spectra

**Figure 5:**
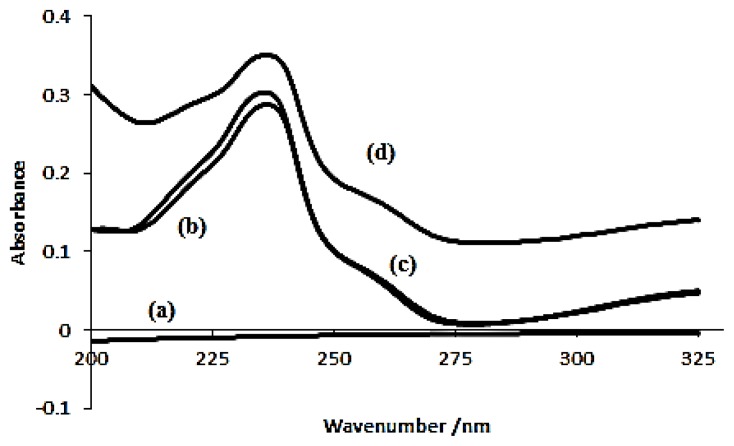
Absorption spectra of (**a**) β-Cyd; (**b**) azomethine; (**c**) physical mixture and (**d**) inclusion complex.

Absorption spectra used to confirm the formation of inclusion complex. In this study, absorption spectra of β-Cyd, azomethine, physical mixture and inclusion complex were taken into consideration [31]. In Figure 5, it was recorded that β-Cyd has almost no absorption throughout the wavelength; hence, its absorbance can be neglected [31]. Absorption spectra for azomethine and the physical mixture were similar at most of the points along the wavelength recorded, which shows the absorbance arises from azomethine alone. Whereas, inclusion complex had an increased intensity at all points of wavelength due to the formation inclusion phenomena between β-Cyd and azomethine. The same phenomena has been observed by Wang *et al.* [32] in their interaction studies of β-Cyd and orange G.

#### 2.2.3. Stoichiometry and Apparent Formation Constant for Azomethine-β-Cyd Inclusion Complex

**Figure 6:**
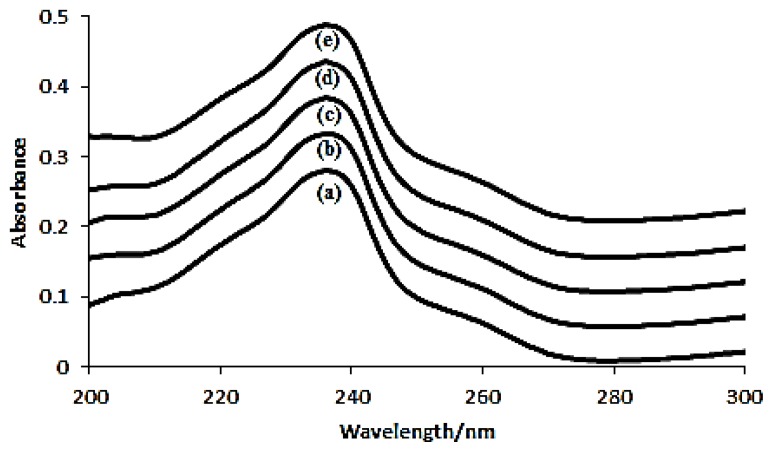
Absorption spectra of azomethine with various concentration of β-Cyd. (**a**) 0 M, (**b**) 3 × 10^−5^ M, (**c**) 4 × 10^−5^ M, (**d**) 5 × 10^−5^M, (**e**) 6 × 10^−5^ M.

To determine the apparent formation constant for the inclusion complex of azomethine and β-Cyd, the concentration of azomethine was held constant at 9 × 10^−3^ M. Meanwhile concentrations of β-Cyd has been manipulated as 0 M, 3 × 10^−5^ M, 4 × 10^−5^ M, 5 × 10^−5^ M and 6 × 10^−5^ M. The absorbance of solutions were measured at 236 nm against a reagent blank which were prepared with identical reagent concentration but without azomethine. The absorption spectra of inclusion complex at different concentrations range of β-Cyd is shown in Figure 6.

**Figure 7:**
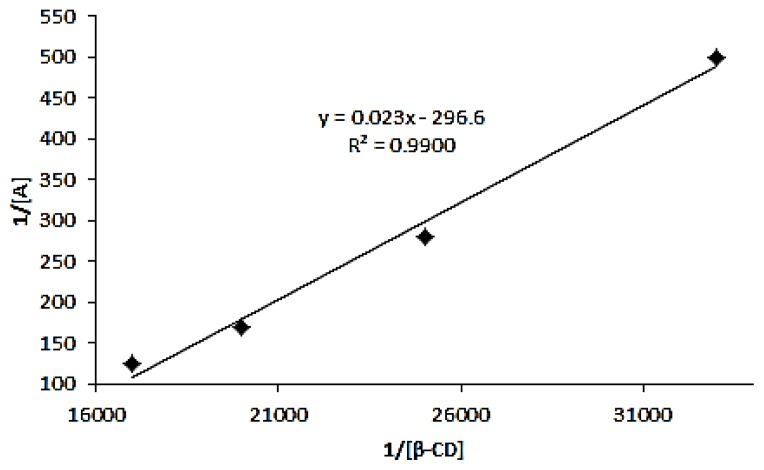
Reciprocal plot for 1/A against 1/[β-Cyd] of azomethine-β-Cyd inclusion complex.

It is observed that, the absorbance value increased with increasing β-Cyd concentration while the concentration of azomethine remains the same. It indicates the solubility of guest molecule (azomethine) increases upon forming the inclusion complex [33]. Since the benzyl part of azomethine more likely to enter the hydrophobic cavity of β-Cyd, stoichiometric ratio of inclusion complex should be therotically 1:1. This theory can be proved if a linear relationship obtained from the reciprocal plot of 1/*A vs.* 1/[β-Cyd] based on the Hildebrand-Benesi Equation 1 [34].

(1) $$\frac{1}{A}=\frac{1}{ɛ{\left[G\right]}_{0}K[CD]}+\frac{1}{ɛ{\left[G\right]}_{0}}$$

where *A* is the absorbance for the azomethine solution at each β-Cyd concentration while [*G*]_0_, *K*, [*CD*], ɛ are the initial concentration of azomethine, apparent formation constant, the concentration of β-cyclodextrin and the molar absorptivity, respectively. Figure 7 shows reciprocal plots that determine the stoichiometry ratio of the inclusion formation. A very good linear relationship was obtained for 1/*A vs.* 1/[β-Cyd] with *R*^2^ = 0.9900. This reciprocal plot clearly indicates the stoichiometry ratio for the inclusion formation between azomethine and β-Cyd is 1:1. The same phenomenon has been observed by Wang *et al.* [32] by using a derivative of sulphonated azo compound like orange *G* to form inclusion complex with β-Cyd.

The apparent formation constant was determined based on the reciprocal plot from Figure 7. With correlation coefficient, *R*^2^ = 0.9900 and the apparent formation constant was 1.29 × 10^4^ L/mol.

## 3. Experimental Section

### 3.1. Reagents and Solutions

Azomethine (Sigma Aldrich, 95%) and β-cyclodextrin (Acros Organics, 99%) were used as received without further purification. Other chemicals such as DMSO, buffer solutions with pH 2, pH 7 and pH 12 were purchased from R & M Chemicals and distilled water was used throughout the research.

### 3.2. Synthesis of the Inclusion Complex of β-Cyd and Azomethine

The inclusion complex of β-Cyd and azomethine was synthesized by using kneading method. Both compounds were mixed in 1:1 mol ratio and grinded thoroughly in a mortar for 30 min and ethanol has been added throughout the grinding process. The precipitate formed was left in oven for drying at temperature of 50 °C for 5 days. Then the precipitate was taken out from the mortar and placed in a vial and kept in a desiccator.

The physical mixture of β-Cyd and azomethine was prepared according to Wang *et al.* [35], where, β-Cyd and azomethine were separately mixed in a mortar. Both compounds were weighted out at a molar ratio of 1:1 and were mixed together until a homogeneous mixture was formed.

### 3.3. Characterizations

Characterization measurements were done with ^1^H NMR, FTIR and TGA. D_2_O was used as solvent for ^1^H NMR (Lambda JEOL 400 Mhz, JEOL (Malaysia), Petaling Jaya, Malaysia) sample preparation. FTIR samples were prepared as KBr pellets, and its spectra determined by Perkin Elmer RX1 FT-IR spectrometer. The thermal analysis was studied by TGA (TA Instruments Q 500, Research Instruments Sdn Bhd, Petaling Jaya, Malaysia).

### 3.4. Spectroscopic Studies

A UV–Visible spectrophotometer (Shimadzu, Kuala Lumpur, Malaysia) with 1 cm quartz cells was used for all the following spectroscopic studies. Absorption spectra were determined between 190 nm and 400 nm.

#### 3.4.1. Effect of pH

Effect of pH for inclusion complex formed was determined by using three different pH buffers. Buffer solutions with pH 2, pH 7 and pH 12 have been used to determine the optimum pH. Subsequently the optimum pH has been used for the absorption spectra and the formation constant.

#### 3.4.2. Absorption Spectra

Absorption spectra were recorded for β-Cyd, azomethine, the inclusion complex and physical mixture.

#### 3.4.3. Formation Constant

The concentrations of β-Cyd were varied in the range of 0–0.0006 M while the concentration of azomethine was held constant at 9 × 10^−3^ M. The data obtained were used to illustrate reciprocal plot for azomethine-β-cyclodextrin inclusion complex with 1/A *vs.* 1/[β-Cyd].

## 4. Conclusions

A novel azomethine-β-Cydinclusion complex was successfully synthesized, and characterized using FTIR, ^1^H NMR and TGA. Azomethine-β-Cydinclusion complex has performed 1:1 host-guest interaction at pH 7 with an apparent formation constant of 1.29 × 10^4^ L/mol which was calculated by Hildebrand-Benesi equation.

## Figures and Tables

**Table 1:** Comparison between the intensity of β-Cyd and the inclusion complex.

Functional Group	Wavenumber (cm^−1^)		Changes Δδ

	β-Cyd	Inclusion complex	
ν[OH] symmetric and antisymmetric	3370.72	3379.88	+9.16
ν[CH_2_]	2928.53	2923.12	−5.53
ν[C–C]	1157.84	1157.66	−0.18
ν[O–H] bending vibration	1029.24	1079.36	+50.12

**Table 2:** Comparison between the intensity of azomethine and the inclusion complex.

Functional Group	Wavenumber (cm^−1^)		Changes Δδ

	Azomethine	Inclusion complex	
ν[OH]	3449.28	3379.88	−69.4
ν[C=N] stretching vibration	1608.52	1639.04	+30.52
ν[=C–H]	681.39	760.28	+78.89

**Table 3:** Chemical shifts (ppm) for the protons of β-cyclodextrin and inclusion complex.

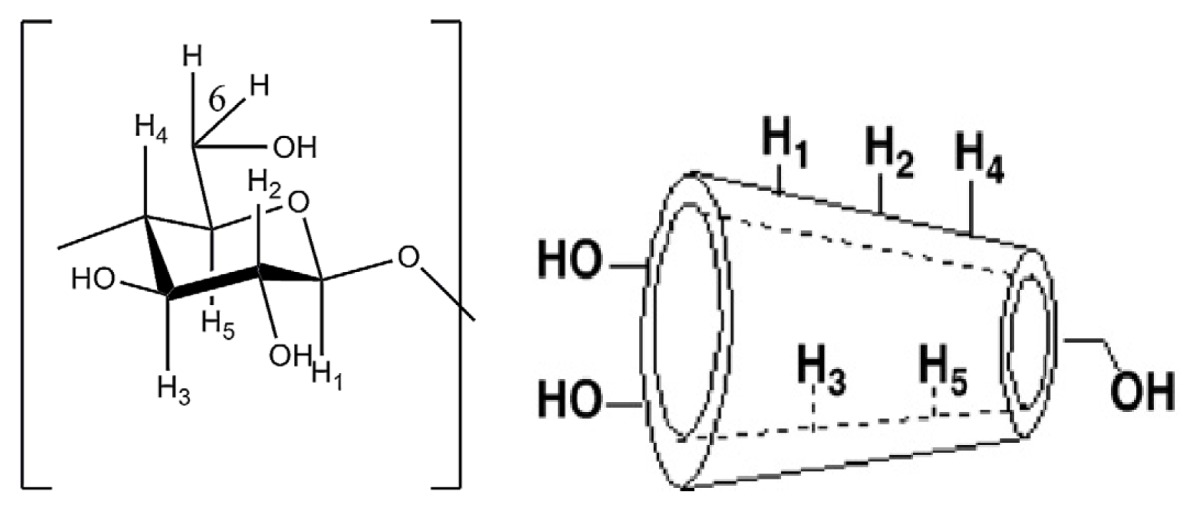						

	H1	H2	H3	H4	H5	H6
β-Cyd 5.0642	3.6309	3.9616	3.5833	3.6650	3.9616	
Inclusion complex	5.0300	3.4400	3.6800	3.4000	3.3900	3.7700
Δδ	0.0342	0.1909	**0.2816**	0.1833	**0.2750**	0.1916
